# Reflections on the early days of the DNA damage response pathway in eukaryotes

**DOI:** 10.1093/genetics/iyag202

**Published:** 2026-10-08

**Authors:** Stephen J Elledge

**Affiliations:** Department of Genetics, Harvard Medical School, Division of Genetics, Department of Medicine, Brigham and Women's Hospital, Howard Hughes Medical Institute, 77 Avenue Louis Pasteur, Boston, MA 02446, United States

**Keywords:** genetics, DNA damage response, checkpoints, damage signaling, kinase cascade

## Abstract

The DNA damage response (DDR) pathway has emerged as the central signaling network that preserves genome stability by using sensors to monitor the genome for aberrant structures and establishing signaling centers that promote organismal homeostasis. This review revisits the formative years of DDR research, focusing on the genetic dissection of the pathway in *Saccharomyces cerevisiae* and its evolution into a broadly conserved eukaryotic signaling system. Early studies of ribonucleotide reductase regulation, replication stress–induced transcription, and cell cycle control identified key regulators, including MEC1, DDC2, TEL1, MRC1, RAD53, DUN1, and RAD9, providing the first evidence that DNA damage signaling is propagated through a protein kinase cascade. This cascade is initiated when RPA binds to ssDNA generated by replication blocks and double-strand DNA breaks, thereby activating the apical MEC1/ATR kinase. These discoveries integrate the contemporaneous cell cycle checkpoint literature into a more comprehensive framework in which the DDR coordinates replication fork stability, replication origin firing, nucleotide metabolism, DNA repair, genome maintenance, and cell cycle control to maintain genomic stability. Parallel studies in *Schizosaccharomyces pombe*, *Xenopus*, and mammalian systems revealed the remarkable evolutionary conservation of the pathway and linked DDR dysfunction to a wide spectrum of human diseases, including cancer. In retrospect, the emergence of the DDR field highlights the extraordinary power of classical genetics combined with molecular biology to uncover a complex signaling network that now occupies a central position in chromosome biology, cancer research, aging, immunity, and therapeutic responses.

## Prelude

I am delighted to write this personal reflection for the journal GENETICS because it has played a key role in publishing some of the early papers on the DNA damage and replication stress response pathway. But far beyond that, genetics as a discipline and as a way of thinking has guided everything I have done, and the dissection of this pathway was a perfect example of how one can use genetics to unravel mystery after mystery of a pathway for which only a glimpse initially existed. I focus on the early work in *S. cerevisiae* from my research perspective, but much work was also done in parallel in *Schizosaccharomyces pombe, Xenopus,* and mammals, which will be referenced only in passing due to space limitations. This article is not meant to be complete, as covering everything is an impossible task. I recommend previous reviews on these areas (Callegari and Kelly 2007; Harper and Elledge 2007; Kerzendorfer and O'Driscoll 2009; Ciccia and Elledge 2010; Smith et al. 2010; Ma et al. 2020; McClure et al. 2022; Groelly et al. 2023)

## Early days: my introduction to DNA damage

From the moment the structure of DNA was revealed to the world in 1953 (Watson and Crick 1953), its inherent beauty was overwhelming. The double helix offered an immediate clue to its own replication, while also providing the first glimpse of the blueprint of life, deeply encoded in a cryptic script that awaited deciphering. And deciphered it was. DNA was the quintessential information molecule, carrying within it a record of 3 billion years of evolution and instruction.

I fell in love with DNA as a senior chemistry major at the University of Illinois. I had avoided biology, dismissing it as “soft” science after uninspiring experiences dissecting frogs in high school. Then, in 1977, I took a biochemistry course as an elective. One lecture, on recombinant DNA and the possibility of rewriting gene function, stopped me in my tracks. I realized this changed everything. Biology had become molecular, and at its center stood the most fascinating molecule imaginable. How could a chemist resist? I quickly abandoned plans to apply to organic chemistry PhD programs and turned instead toward molecular biology, a path that led me to the Department of Biology at the Massachusetts Institute of Technology. It was one of several turns in my life that proved far more rewarding than I could ever have imagined.

Having never performed research or taken a proper biology course, I suddenly found myself in the Mecca of molecular biology—and well behind my entire class. To catch up, I immersed myself in the fundamentals by taking 13 full-semester courses, ranging from molecular biology and cell biology to phage and bacterial genetics, virology, and immunology. That intensive foundation went on to shape my entire future research trajectory.

For my PhD, I was fortunate to join the lab of Dr. Graham Walker, also a chemist by training and a wonderful mentor, who was studying mutagenesis in *Escherichia coli*. At the time, a paradox had emerged: UV-induced mutagenesis in *E. coli* was not simply a passive process, but instead required the *E. coli umuC* gene (Kato and Shinoura 1977). I cloned the *umuC* locus, and found it encoded 2 proteins, UmuC and UmuD (Elledge and Walker 1983), which Hatch Echols’ lab showed comprised the first error-prone DNA polymerase, the *Pol V* family (Rajagopalan et al. 1992). During this period, I learned of the pioneering work of Evelyn Witkin, Miroslav Radman, Graham Walker, David Mount, Jeff Roberts, Raymond Devoret, and others, who were elucidating cellular responses to DNA damage that came to be known collectively as the SOS response in bacteria (reviewed in Witkin 1991; Smith and Walker 1998). In this response, a set of repressors, e.g. LexA and Lambda cI, had evolved autoprotease sites that could be activated by sensing active homologous recombinational repair by the RecA protein when cells encountered DNA damage. The inactivation of LexA turned on a number of genes involved in DNA repair, such as excision repair proteins, error-prone polymerases like UmuDC, and RecA itself. Lambda cI inactivation allowed the lysogenic lambda phage to become lytic. This regulatory relationship was the first documented pathway for regulated responses to DNA damage.

## Accidents will happen; be prepared

I left DNA repair research to move into eukaryotic biology, joining Ron Davis's lab as he began exploring plant systems. Ron was an inventor at heart, with a deep enthusiasm for technology and method development. That mindset resonated with me. I had always enjoyed developing new cloning methods as a graduate student, and before long, I drifted away from plant biology toward considering what abilities science lacked. It seemed obvious to me that we needed a way to knock out genes in mammalian cells using homologous recombination. My initial idea was straightforward: purify mammalian recombinases, coat DNA substrates with them, and introduce these complexes into cells to drive targeted integration. One obstacle stood in the way: no mammalian RecA homolog had yet been identified. I reasoned that I could first isolate the budding yeast homolog and then use its sequence to find the mammalian gene. Using antibodies to bacterial RecA, I screened yeast extracts by western blot and identified a candidate protein of the expected size. Encouragingly, it also appeared to be induced by DNA damage. I then set out to clone the gene using the λgt11 expression system developed in the Davis lab, and I succeeded in isolating a clone.

The excitement was short-lived. DNA sequencing revealed no homology to RecA, except that the last 4 amino acids were identical and were, in fact, the cross-reacting epitope. Despite the very limited sequence databases available in 1984, there was a clear match to a known protein in mammals and clams: the small subunit of ribonucleotide reductase, the enzyme responsible for generating deoxyribonucleotides for DNA synthesis. I was devastated. Knowing when to drop a project is as important in science as having a good idea, so I set this aside to explore other avenues.

## Following the regulation

A turning point came a year later, when David Stillman visited Stanford for a job interview. During our conversation, David mentioned that ribonucleotide reductase was particularly interesting because its abundance and activity were regulated during the mammalian cell cycle. That prompted me to think more carefully about the gene I now call *RNR2*. I decided to characterize it further and eventually wrote it up (Elledge and Davis 1987). In the discussion section of that paper, my earlier exposure to the *E. coli* SOS response prepared me for what I might have stumbled upon in eukaryotes. I suggested that analysis of this pathway could reveal both the signal generated by DNA damage and the proteins responsible for transmitting that signal to the *RNR2* promoter. I referred to this process as “signal transduction,” but the reviewers insisted that I remove the term, arguing that signal transduction applied only to signals traveling from outside the cell to the inside. Early days.

I found *RNR2* inducibility to be cell autonomous, independent of protein synthesis, and that blocking DNA replication with hydroxyurea (HU), which does not itself cause DNA damage, produced the strongest induction of *RNR2* expression. This observation implied that the cell might be sensing the status of DNA replication itself (Elledge and Davis 1989a). I identified a repressor element that appeared to mediate this regulation (Elledge and Davis 1989b). I went on to identify the genes encoding the large subunits of ribonucleotide reductase and found 2: *RNR1*, an essential gene that is tightly regulated during the cell cycle, and *RNR3*, which is normally silent but can be induced up to 100-fold in response to DNA replication stress (Elledge and Davis 1990). This stringent regulation made the system especially powerful for genetic screens.

While I was working out the *RNR* family and regulatory characteristics, an important paper from the lab of the renowned geneticist Lee Hartwell (Weinert and Hartwell 1988) described that an existing radiosensitive mutant in budding yeast, *rad9*, was defective in cell cycle arrest in response to ionizing radiation. In this study, they coined the “checkpoint” concept, in which cell cycle transitions depend on the completion of prior events enforced by inhibitory elements. This was elaborated upon in a subsequent review (Hartwell and Weinert 1989). Beyond its huge conceptual impact on the cell cycle field, the cell cycle response was important for 2 practical reasons. First, it provided a second assay for the response to damage, which proved useful in future analyses as it was likely to be involved with signal transduction. Second, because the cell cycle field was large, it attracted considerable attention and drew researchers into the field. Ironically, despite the galvanizing effect that paper had on the cell cycle field, the observation that multiple radiation-sensitive mutants in fission yeast failed to delay the cell cycle after irradiation, including *rad3*, *rad1* and *rad9*, was published 12 years earlier by Nasim (Hannan et al. 1976). (It should be noted that the *RAD* genes in budding and fission yeast have different numbering, a source of endless confusion.) Nasim’s paper was overlooked for many years, possibly because the cell cycle field was not as advanced in 1977. Consequently, the paper's significance was not immediately recognized nor appropriately referenced thereafter.

## Letting genetics lead the way

After establishing my lab at the Baylor College of Medicine, my first graduate student, Zheng Zhou, and I set out to develop a screen to identify regulators of this pathway, a project that led to 2 of my favorite papers. The screen employed 2 reporters driven by the *RNR3* promoter: one controlling the *URA3* coding sequence for selection and the other *lacZ* for quantitative measurement. Our first paper, published in *GENETICS* (Zhou and Elledge 1992), described a screen designed to isolate spontaneously arising constitutive mutants of *RNR3*, with the goal of identifying the signals sensed by this pathway. From 202 mutants exhibiting the URA^+^ phenotype across 2 mating types, we identified 200 recessive *crt* (constitutive *RNR3* transcription) mutants, which mapped to 9 complementation groups.

Further analysis revealed that the *crt* mutants fell into 2 categories (Fig. 1a). Class I genes were involved in DNA replication and included *CRT5 (POL1/CDC17)* and 3 ribonucleotide reductase subunits: *CRT3 (RNR4), CRT6 (RNR2)*, and *CRT7 (RNR1)*. Class II genes functioned in transcriptional repression and included *CRT4 (TUP1)* and *CRT8 (SSN6)*. Perhaps most importantly, the screen uncovered a viable mutation in the replicative DNA polymerase α (*POL1/CDC17*), which initiates DNA synthesis on both the leading and lagging strands at the replication fork. This finding suggested that signaling may originate directly from the replication fork, consistent with observations from HU treatment and mutations in ribonucleotide reductase subunits. Together, these insights strongly supported the idea that this pathway was intimately linked to DNA replication.

**Fig. 1.**
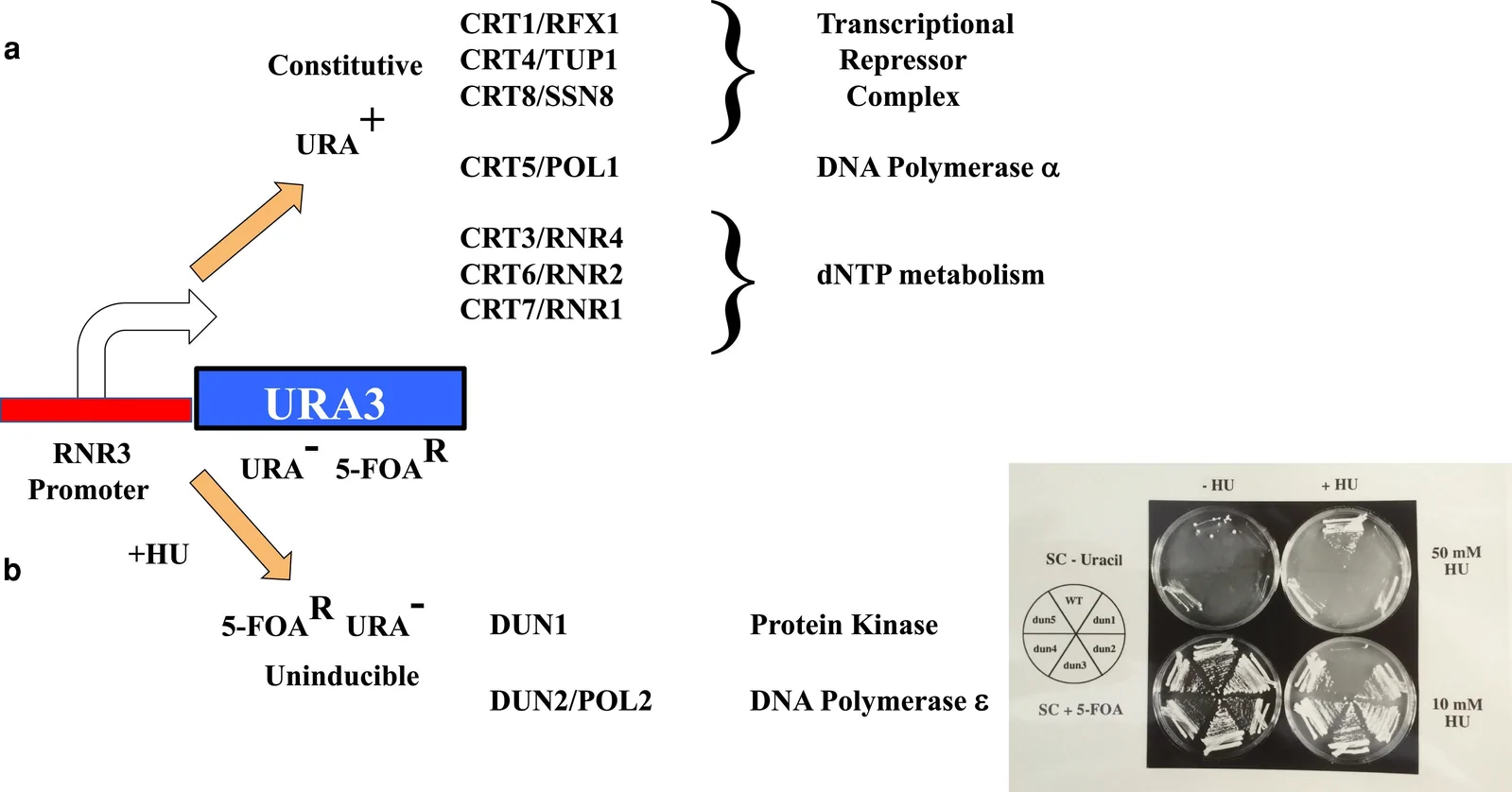
Screens for regulators of the DNA replication stress response pathway controlling the *RNR3* promoter. a) Outline of the *CRT* (Constitutive *RNR3* Transcription) screen. Spontaneous constitutive *URA*^+^ mutants were examined and fell into 9 complementation groups, of which the genes responsible for 7 are shown. b) Outline of the *DUN* (DNA Damage Uninducible) screen, which revealed 5 complementation groups, and the genes encoding 2 of them are shown. The yeast strains with genotypes indicated, and their growth phenotypes on the indicated media are illustrated on the right, reprinted with permission from Cell Press (Zhou et al. 1993).

The use of the *URA3* gene as a reporter for *RNR3* expression was deliberate, as it enables both positive and negative selection. In the presence of 5-fluoro-orotic acid (5-FOA), *URA3* expression becomes toxic, allowing selection for mutants defective in *RNR3* inducibility. *URA3* encodes orotidine-5′-phosphate decarboxylase, a key enzyme in uracil biosynthesis that converts 5-FOA into 5-fluorouracil (5-FU). This metabolite is further processed into FdUMP, an inhibitor of thymidylate synthetase, thereby blocking dTTP synthesis and ultimately DNA replication.

We reasoned that even low levels of *RNR3*-driven *URA3* expression induced by a small amount of HU would, in the presence of 5-FOA, amplify replication stress. This creates a feedback loop in which inhibition of DNA synthesis further enhances *RNR3* induction, driving lethal *URA3* expression unless the cell is defective in *RNR3* inducibility. By plating cells on media containing low concentrations of both HU and 5-FOA, we were able to selectively isolate DNA damage uninducible (*dun*) mutants with defects in this signaling pathway. Sixteen *dun* mutants formed 5 complementation classes, with 12 mutations mapping to the *dun1* group (Zhou and Elledge 1993) (Fig. 1b). Life had given me lemons, and genetics made lemonade.

## Establishing the signal transduction pathway

Cloning of *DUN1* revealed that it encodes a protein kinase whose activity increases in response to DNA damage. Deletion of *DUN1* or mutation of its catalytic site abolished the inducibility of all *RNR* genes. Together, these findings provided the first strong evidence that the DNA damage response (DDR) operates through a regulated signal transduction pathway.

The identification of a signaling pathway and multiple participating genes led us to focus on 3 distinct questions: (i) Which substrates does Dun1 phosphorylate to regulate transcription? (ii) What acts upstream of Dun1 to mediate its activation? (iii) What is the sensor that detects replication fork stress signal?

The *GENETICS* paper (Zhou and Elledge 1992) provided the critical clue to answering the first question. We found that *crt1* mutations were epistatic to *dun1* mutations and caused constitutive *RNR3* expression. *CRT1* encoded a transcriptional repressor that bound the promoters of all *RNR* genes and recruited the co-repressors Crt4 (Tup1) and Crt8 (Ssn6). In response to DNA damage, Crt1 became phosphorylated, disrupting its DNA-binding activity and thereby derepressing *RNR* transcription. Crt1 also repressed its own promoter, so *CRT1* transcription was induced together with the *RNR* genes, creating a negative feedback loop that shut down the response once the inducing signal subsided (Huang et al. 1998). In this way, the mechanism underlying transcriptional induction was elucidated.

Next, the upstream regulators of *DUN1* were explored by screening for *SAD* (S-phase arrest-defective) mutants. The rationale was that mutants in the genes upstream of *DUN1* would also be sensitive to HU. We screened them for defects in *RNR3* transcriptional induction and also considered that, if this pathway also controlled cell cycle arrest in S phase, they might show signs of cell cycle progression, such as elongated spindles indicative of mitotic entry. Two of 5 mutants were defective for *RNR* transcription, *sad1* and *sad3* (Allen et al. 1994), and *sad1* mutants were allelic with *rad53* mutants. A parallel study (Weinert et al. 1994) also identified mutants in *RAD53* and in a gene dubbed *MEC1*, which was allelic to *SAD3*. We cloned *RAD53*, which revealed that it was also a protein kinase whose kinase activity was required for the phosphorylation and activation of the *DUN1* kinase. Unexpectedly, *RAD53* was an essential gene, contrary to the prevailing notion at the time that “checkpoint” pathways were layered on top of cell cycle regulatory pathways and came into play only when damage occurred. This meant *RAD53* must control more than *DUN1*, and that this regulatory pathway is activated in every cell cycle, not only when damage is present. Parallel studies in *S. pombe* genetically identified several homologous core elements that were partially overlapping with the original Nasim *RAD* mutants (Hannan et al. 1976; al-Khodairy and Carr 1992; Enoch et al. 1992; al-Khodairy et al. 1994; Murakami and Okayama 1995). Thus, we had established what appeared to be a protein kinase cascade, further supporting the signal transduction aspects of the pathway, but raising additional questions, such as what acts upstream and what the pathway's essential function is. Another DDR-responsive kinase, Chk1, was originally discovered in *S. pombe*, and we found the *S. cerevisiae* ortholog to work in a signaling branch parallel to Rad53 (Walworth et al. 1993; Sanchez et al. 1997; Sanchez et al. 1999). To help avoid confusion caused by different gene names across species, the outline of the signal transduction pathway is shown, with orthologous gene names in different species in Fig. 2.

**Fig. 2.**
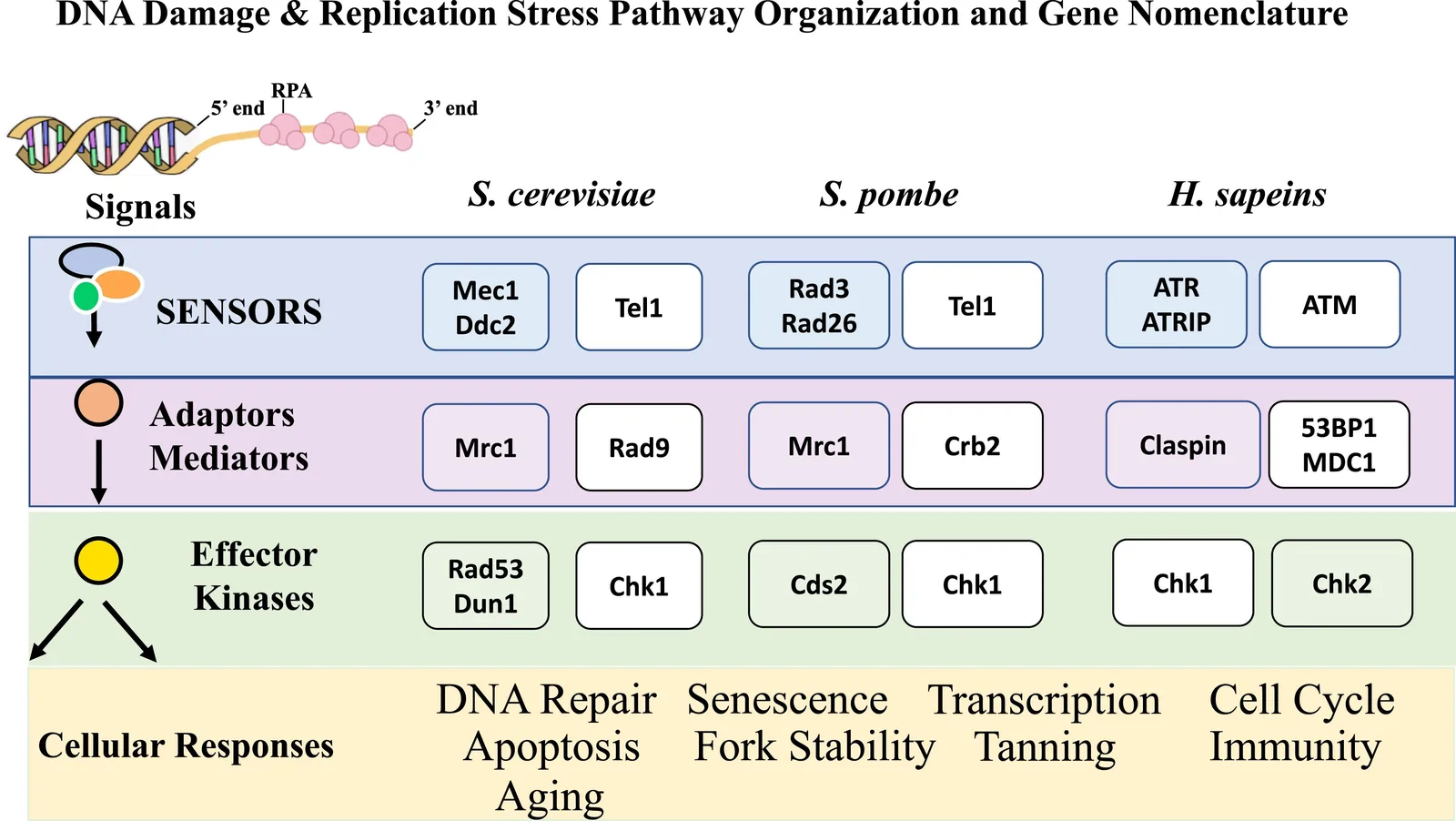
The overall structure of the DNA Damage and Replication Response signal transduction pathway in *S. cerevisiae* is shown, and the orthologous genes in *S. pombe* and *Homo sapiens* are indicated along with examples of cellular responses across all species. Boxes with similar colors are the orthologs across rows. Rad53 and Dun1 are shown together because they are both members of the FHA domain kinase family, along with Cds2 and Chk2. For simplicity, the *MRN* complex (Carney et al. 1998) for ATM/TEL1 DNA sensing, and the Rad17 and 911 complexes and the *ATR/MEC1* activators *DPB11*, *TOPBP1* and *ETAA* described below are not shown.

Considering what might be upstream of *RAD53*, we reasoned that the regulator would also be an essential gene and might be suppressible by overproducing *RAD53*. We searched for *rod* (Rad53-overproduction-dependent) mutants and found one that was allelic to *SAD3/MEC1*. We discovered that Rad53, like Dun1, was phosphorylated in response to replication stress and that this phosphorylation was dependent on *MEC1/SAD3* function, thereby placing it upstream of *RAD53* (Sanchez et al. 1996). This was the time of the yeast genome project, and after cloning *MEC1*, it was also found to be a protein kinase of the PI3K-like family and was previously identified and cloned as a meiotic regulator, ESR1 (Kato and Ogawa 1994). We found a related gene, *TEL1* (Lustig and Petes 1986), in the database and showed that it was partially redundant with *MEC1*, as did the Heiter lab (Morrow et al. 1995), and that its overproduction could also suppress *mec1* null mutants and weakly activate Rad53. *MEC1* was also related to the fission yeast *RAD3* gene, (Seaton et al. 1992) and its human homolog was named *ATR* (Bentley et al. 1996; Cimprich et al. 1996). Likewise, a *TEL1*-related gene called *ATM* was cloned (Savitsky et al. 1995) and was responsible for the disease ataxia telangiectasia. Cells derived from these patients were radiation sensitive (Painter and Young 1980). So, not only was the pathway taking shape as an even larger kinase cascade but its conservation across multiple species was coming into view (Fig. 2). These were exciting times.

## Is the checkpoint really the point?

The checkpoint concept had a profound impact on the cell cycle field by establishing the idea that a network of negative regulators imposes order on cell cycle transitions. At the time, the prevailing view in the field was that the primary function of this signal transduction pathway was simply to arrest the cell cycle in response to DNA damage. This interpretation became so deeply embedded in the field that it was difficult to convince researchers that, unlike *RAD9*, this pathway governed a much broader physiological response, one that coordinated not only cell cycle arrest but also the maintenance of replication fork homeostasis under conditions of replicative stress.

Several observations suggested that the “cell cycle arrest” model was incomplete. First, the replication stress response pathway clearly played a positive regulatory role in promoting DNA replication by stimulating nucleotide synthesis. Second, as noted above, *MEC1* and *RAD53* are essential genes, contradicting the widely held notion that these pathways are engaged only under conditions of DNA damage.

A clearer understanding emerged in part from studies by Brian Desany in my laboratory. He demonstrated that the lethality of *rad53* and *mec1* null mutations could be rescued by overexpression of *RNR1*, which encodes the rate-limiting enzyme in dNTP synthesis. In parallel, transient treatment of *mec1* or *rad53* mutant cells with HU as they entered the S phase caused irreversible cell death that could not be prevented by blocking mitotic entry with microtubule poisons. Moreover, cells subjected to only a 30-min inhibition of DNA replication failed to complete genome replication, as shown by pulsed-field gel electrophoresis of whole chromosomes (Desany et al. 1998). Related work demonstrated that replication forks collapse under similar conditions (Lopes et al. 2001). This also likely explained the observations in *S. pombe* that the cell cycle checkpoint *hus-1* mutants died faster than the *cdc2-w* checkpoint mutants in the presence of HU (Enoch et al. 1992). Furthermore, the apparent entry into anaphase observed in *rad53* mutants in the presence of HU, indicated by spindle elongation, was actually due to a failure to properly replicate centromeres rather than to cell cycle progression (Julius et al. 2019).

Important physiological connections came from the lab of John Diffley, who gained a much greater appreciation for the intimate role of this pathway in coordinating DNA replication and replication fork stability. They showed that *MEC1* and *RAD53* inhibit late origin firing in response to replication stress by phosphorylating Sld3 and Sld4 (Santocanale and Diffley 1998; Shirahige et al. 1998; Zegerman and Diffley 2010). They also showed that phosphorylation of Mrc1, described below, slowed fork progression in the presence of DNA damage (Tercero and Diffley 2001; McClure and Diffley 2021). Together, these findings established that the essential function of this pathway is not merely to halt the cell cycle but to stabilize replication forks and sustain DNA synthesis during the S phase.

## What is sensing the damage signal? First, a red herring

We obtained our first hint of a possible sensor for the replication stress response from the *DUN* screen. One of the genes identified, *DUN2*, encoded DNA polymerase ε (*POL2*), the leading-strand DNA polymerase positioned directly at the replication fork (Navas et al. 1995). This localization raised the intriguing possibility that DNA polymerase ε itself might participate in sensing replication stress. We showed that *POL2* and *RAD9* controlled distinct branches of the checkpoint response (Navas et al. 1996), further supporting the idea that *POL2* might play a direct signaling role.

Subsequent work, however, pointed to *MRC1* as the more likely explanation for the checkpoint defect (Alcasabas et al. 2001). *MRC1*, a nonessential gene whose mammalian homolog is Claspin (*CLSPN*) (Kumagai and Dunphy 2000), was identified in a screen for HU-sensitive mutants required for Rad53 activation. Like *POL2*, it functioned in parallel with *RAD9*. Mrc1 was heavily phosphorylated in an *MEC1*-dependent manner, suggesting that it was a direct target of the DDR signaling. *MRC1* null mutants exhibited slow DNA replication and accumulated DNA damage during the S phase. We found that Mrc1 traveled with the replication fork and became hyperphosphorylated on SQ motifs, the preferred phosphorylation sites for the Mec1/Atr and Tel1/Atm kinases. Mutation of all 17 SQ motifs abolished both Mrc1 phosphorylation and Rad53 activation, while preserving Mrc1's association with the replication fork and its role in DNA replication itself (Osborn and Elledge 2003). Thus, the replication and checkpoint signaling functions of Mrc1 could be genetically separated.

Importantly, the Campbell lab (Lou et al. 2008) demonstrated that Mrc1 physically associates with DNA polymerase ε and that multiple *pol2* alleles exhibiting the *dun*^−^ phenotype produced signaling defects nearly identical to those of *mrc1* mutants. Together, these findings strongly suggested that *POL2* contributes to Rad53 activation indirectly through its functional and physical association with Mrc1. The moral of this story is that while genetics never lies, it can certainly mislead. In this case, *POL2* looked like a duck and quacked like a duck, but ultimately turned out to be a goose.

## The actual signal and the sensor

What then is the signal that is sensed? The solution this time did not emerge from genetics but from metazoan biochemistry as we focused on the apex of the signaling cascade, MEC1/ATR, as the most likely candidate. We found, through purification of ATR, an ATR-binding partner we called ATRIP (Cortez et al. 2001). *ATRIP* was required for *ATR* function, and the fission and budding yeast orthologs, *RAD26* (Edwards et al. 1999) and *DDC2/LCD1*(Paciotti et al. 2000; Rouse and Jackson 2000), were also implicated in DDR function but had no known biochemical function. Lee Zou, who had trained with the superb biochemist Bruce Stillman, began to look for a function. We considered the possibility that it bound ssDNA, as it was associated with all types of DNA damage and its presence correlated with cell cycle arrest (Garvik et al. 1995; Lee et al. 1998). However, it did not. We then considered that ssDNA was unlikely to be naked in cells. It was more likely to be bound by ssDNA-binding proteins like RPA. We tried coating ssDNA with RPA, and presto, ATRIP bound tightly to the RPA-ssDNA structure. Since RNAi was now available, we used it to reduce RPA70 protein levels and observed that in RPA70-depleted cells, UV light–induced ATR-ATRIP foci were significantly reduced, indicating that endogenous RPA-ssDNA was required for signaling (Zou and Elledge 2003).

This, however, was only part of the story. A parallel protein complex involved in sensing replication stress received comparatively less attention: the Rad17 clamp loader complex (Griffiths et al. 1995) and the 9-1-1 (Rad9-Rad1-Hus1) PCNA-like complex (Thelen et al. 1999; Majka et al. 2003), named after the *S. pombe* genes. Rad17 forms a complex with RFC2, RFC3, RFC4, and RFC5 and was thought to function analogously to the RFC1 clamp loader complex. However, rather than loading PCNA trimers, it loads the related 9-1-1 complex. ATR and RAD17 localized independently to replication stress foci, and RAD17 was phosphorylated by ATR *in vivo* (Bao et al. 2001; Kondo et al. 2001; Melo et al. 2001; Zou et al. 2002).

*In vitro*, we found that the addition of ATR/ATRIP, the RAD17 complex, and ssDNA alone did not result in RAD17 phosphorylation (Zou and Elledge 2003). However, inclusion of RPA enabled ATR-dependent phosphorylation of RAD17. What, then, induces the RAD17 complex to load 911, which was also critical for signaling? Once again, the answer was RPA-coated ssDNA and a 5’ ssDNA–dsDNA junction, as demonstrated independently by Stillman’s and our labs (Ellison and Stillman 2003; Zou et al. 2003). Subsequent studies revealed an additional important layer of regulation: the 9-1-1 complex recruits ATR activators, such as TOPBP1/DPB11 and ETAA1, thereby further promoting the assembly and activation of a robust signaling apparatus (Ma et al. 2020). Thus, the juxtaposition and interaction of 2 sensors were required to initiate replication stress signaling. Presumably, the cell avoids spurious signaling because of the profound regulatory consequences that ensue. For example, R-loops could generate RPA-bound ssDNA, but without a free ssDNA–dsDNA junction, it would not activate the signaling cascade.

A second, partially overlapping pathway evolved to sense DSBs in mammals, the ATM-MRN complex (Kastan et al. 1992; Stewart et al. 1999) which phosphorylates the Chk2 kinase (RAD53/DUN1 ortholog) to activate p53 transcription (Matsuoka, et al. 1998; Blasina et al. 1999; Chehab et al. 2000; Hirao et al. 2000; Shieh et al. 2000) along with many other substrates that modulate DNA repair. Together, ATM and ATR sense and coordinate the response to structural alterations in the genome to maintain genomic stability.

The comparison between fungal and mammalian DDR pathways has provided important insights into the evolution of genome surveillance mechanisms. Although the overall architecture of the DDR signaling network as a protein kinase cascade, with mediator proteins serving as signaling intermediaries, is highly conserved, the mammalian pathway is considerably more elaborate. This increased complexity is consistent with the larger and more intricate mammalian genome, higher-order chromosomal organization, extensive heterochromatin, and the additional requirement to coordinate extracellular signaling in multicellular organisms. In fungi, TEL1 shares some functions with MEC1 in the DDR but plays a relatively minor role overall. By contrast, its mammalian homolog, ATM, is a central regulator of the rapid response to DNA double-strand breaks (DSBs). Another key distinction lies in the downstream checkpoint kinases. In fungi, the RAD53/CDS1 pathway is the primary mediator of the replication stress response, whereas its mammalian counterpart, CHK2, functions predominantly with ATM in the response to DSBs. Instead, replication stress signaling in mammals is largely controlled by the CHK1 pathway. The evolutionary pressures that drove this apparent reassignment of checkpoint kinase functions remain an intriguing and unresolved question.

## Conclusion

Since 1992, more than 20,000 papers have been published with “DNA damage response or DNA damage signaling” in the title or abstract, along with an additional 6,000 that refer to “cell cycle checkpoints or DNA damage checkpoints.” In mammals, the DDR mutants have emerged as both a driver of tumorigenesis, through genes such as ATR, ATM, CHK1, CHK2, TP53, PALB2, BRCA1, and BRCA2, and, when WT, a central determinant of chemotherapeutic efficacy, and a target for therapeutic inhibition. Beyond cancer, DDR signaling plays critical roles in brain development, neurodegeneration, hematopoietic disorders, antiviral defense, antigen receptor generation in B and T lymphocytes, apoptosis, senescence, development, chromatin remodeling, genomic stability, inflammation, aging, stem cell maintenance, and immune signaling.

At the molecular level, the DDR kinase cascade induces phosphorylation of more than 1,000 distinct proteins in response to DNA damage (Matsuoka et al. 2007; Bennetzen et al. 2010), thereby extensively reprogramming cellular physiology. Perhaps most striking is the degree to which DDR signaling orchestrates DNA replication dynamics, promotes nucleotide excision repair, R-loop processing, replication fork protection and restart, repression of late origin firing, and coordinates diverse DNA repair machineries. The DDR governs the translesion synthesis pathway through Pol eta, the Fanconi anemia interstrand crosslink repair pathway through FANCI and D2 phosphorylation, and regulates homologous recombination at both the initiation through BRCA1 and BRCA2, and resolution stages through nucleases and scaffold complexes such as SLX4. In effect, the DDR functions as the command-and-control system, sitting at the stalled fork directing biochemical traffic for the intricate replication fork transactions that occur during the replication of the exceptionally complex chromatin environment of mammalian cells.

Looking back on it all, it is remarkable that such a complex signaling network could be dissected within the span of a little more than 15 years—from the first tantalizing glimpses to a coherent conceptual framework spanning organisms from yeast to mammals. Equally remarkable is the realization that this system appears to have evolved twice: once in a relatively compact form in bacteria and again in a distinct, vastly more elaborate, multilayered form in eukaryotes. The field has grown tremendously in the last 15 years and has made many important contributions not covered here.

Of course, these advances represent only the broad outlines. The underlying mechanisms are still being resolved through increasingly sophisticated reconstituted DNA replication systems, deep biochemical analysis, and structural biology, or, increasingly, its AI-enabled counterpart, AlphaFold. Revisiting the early papers can be both inspiring and humbling. One sees the excitement of discovery, but also the inevitable flaws: misinterpretations, incomplete controls, missed connections, and experimental artifacts that, in hindsight, concealed clues that might have accelerated progress by years.

Yet taken together, the story stands as a tribute to the extraordinary power of genetics. From a small collection of chance observations emerged the foundation of a profound signaling pathway, one that is indispensable for life itself and that governs an astonishing range of cellular and organismal processes critical for human health.
